# Nuclear Quantum Effects in Proton or Hydrogen Transfer

**DOI:** 10.1021/acs.jpclett.3c03368

**Published:** 2024-01-10

**Authors:** Jacek Waluk

**Affiliations:** † Institute of Physical Chemistry, Polish Academy of Sciences, Kasprzaka 44/52, 01-224 Warsaw, Poland; ‡ Faculty of Mathematics and Science, Cardinal Stefan Wyszyński University, Dewajtis 5, 01-815 Warsaw, Poland

## Abstract

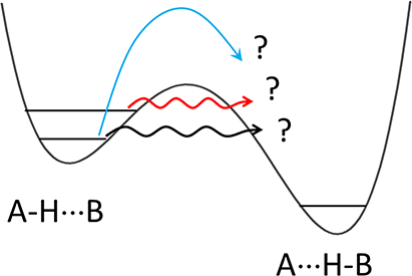

Proton or hydrogen transfers, basic chemical reactions, proceed either by thermally activated barrier crossing or via tunneling. Studies of molecules undergoing single or double proton or hydrogen transfer in the ground or excited electronic state reveal that tunneling can dominate under conditions usually considered to favor the thermal process. Moreover, the tunneling probability strongly varies for excitation of certain vibrational modes, which changes the effective barrier and/or proton transfer distance. When the reaction is fast compared to vibrational relaxation, the mode selectivity can still be maintained for molecules in solutions at 293 K. These observations point to dangers of relating the calculated minimum energy paths and the associated barriers to the experimentally obtained activation energies. The multidimensional character of the reaction coordinate is obvious; it can dramatically change for slowly and rapidly relaxing environments. We postulate that the hydrogen bond definition should be extended by specifically including the role of molecular vibrations.

One of the fascinating consequences of quantum mechanics is the phenomenon of tunneling: crossing an energy barrier without energy investment.^1^ Such passage may deviate from the minimum energy path, because, unlike in Arrhenius equation, the probability of tunneling can be expressed as *P* = ,^2^ where *E* is the barrier height, *m* is the mass of the tunneling particle, *h* is Planck’s constant, and *w* is the barrier width. The latter parameter is responsible for “corner cutting”: the system selects a shorter path, even though it implies a higher barrier.

During the nearly 100 years that have passed since tunneling was first discussed,^1^ the appreciation of its role in chemical reactions changed dramatically. Initially, it was considered as dominant exclusively at cryogenic temperatures and involving only light particles: electrons and protons. However, over the last decades it became obvious that heavier elements such as carbon can also tunnel^2,3^ and that tunneling can be important even above room temperature, as demonstrated recently for hydrogen.^4^

Proton or hydrogen transfer, a ubiquitous and seemingly simple chemical transformation, is, in fact, one of the most complicated reactions. This is due to the quantum nature of the proton coupled with its small mass, a combination that can make tunneling highly probable. Moreover, the tunneling probability may be very different for various vibrational modes. It can also be dramatically influenced by the environment. In addition, distance dependence of the tunneling rate is much stronger for protons than for electrons, since the nuclear wave function is much more localized. All of these factors make it extremely difficult to correctly describe the reaction mechanism: a one-dimensional reaction path is not only too simplistic, but it can also, in certain cases, be misleading. A correct description requires a multidimensional approach with both intra- and intermolecular degrees of freedom properly taken into account. This is illustrated in Figure 1. A simple 1D model suggests that adding energy to the reactant should result in a larger reaction rate since the system comes closer to the top of the barrier. However, this would be the case only if the reaction path coincided with one of the vibrational modes. Otherwise, putting the energy into a specific mode can lead, depending on the character of the vibration, to a decrease or increase of the effective barrier “seen“ by the tunneling particle. A third possibility, lack of response, indicates that the excited vibration is orthogonal to the reaction path.

**Figure 1:**
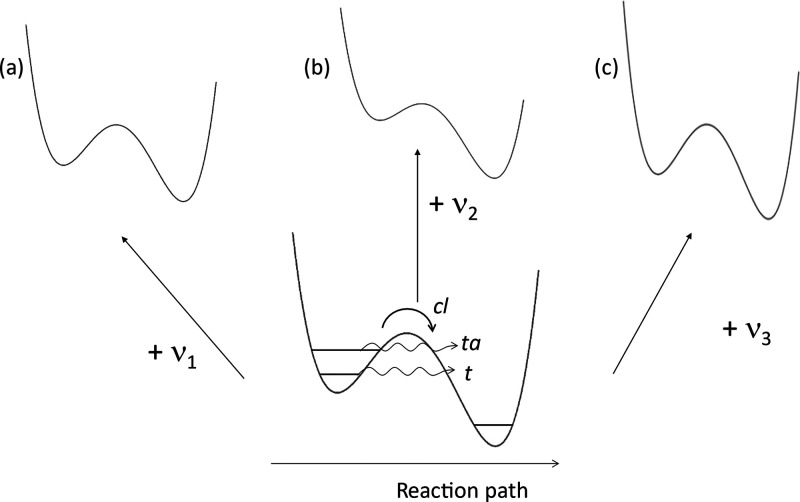
Bottom: 1D representation of a reaction coordinate. Possible barrier crossing paths: tunneling from a vibrationless level (*t*, deep tunneling), thermally (vibrationally) activated tunneling (*ta*), and classical, over the barrier passage (*cl*). Top: Effective potentials “felt” by the tunneling particle when a certain molecular vibration is excited: (a) no change in the potential; (b) barrier decrease; (c) barrier increase. For cases b and c, a change of the barrier width is also expected.

In this Perspective, several examples of nontrivial consequences of tunneling will be presented, for which the analysis of the experimental data allowed understanding of such rather unusual observations as (i) complete disagreement between the calculated reaction barrier and experimentally found activation energy; (ii) vibrational mode-selective tautomerization; (iii) dominant role of tunneling even at room temperature; (iv) kinetic preference for a double over a single hydrogen transfer, in disagreement with calculated barriers; and (v) huge changes in tautomerization rates caused by different time scales of environment dynamics.

## Vibrational Mode-Selective Proton or Hydrogen Transfer

### 2,2′-(Pyridyl)pyrrole (PP)

**Figure 2:**
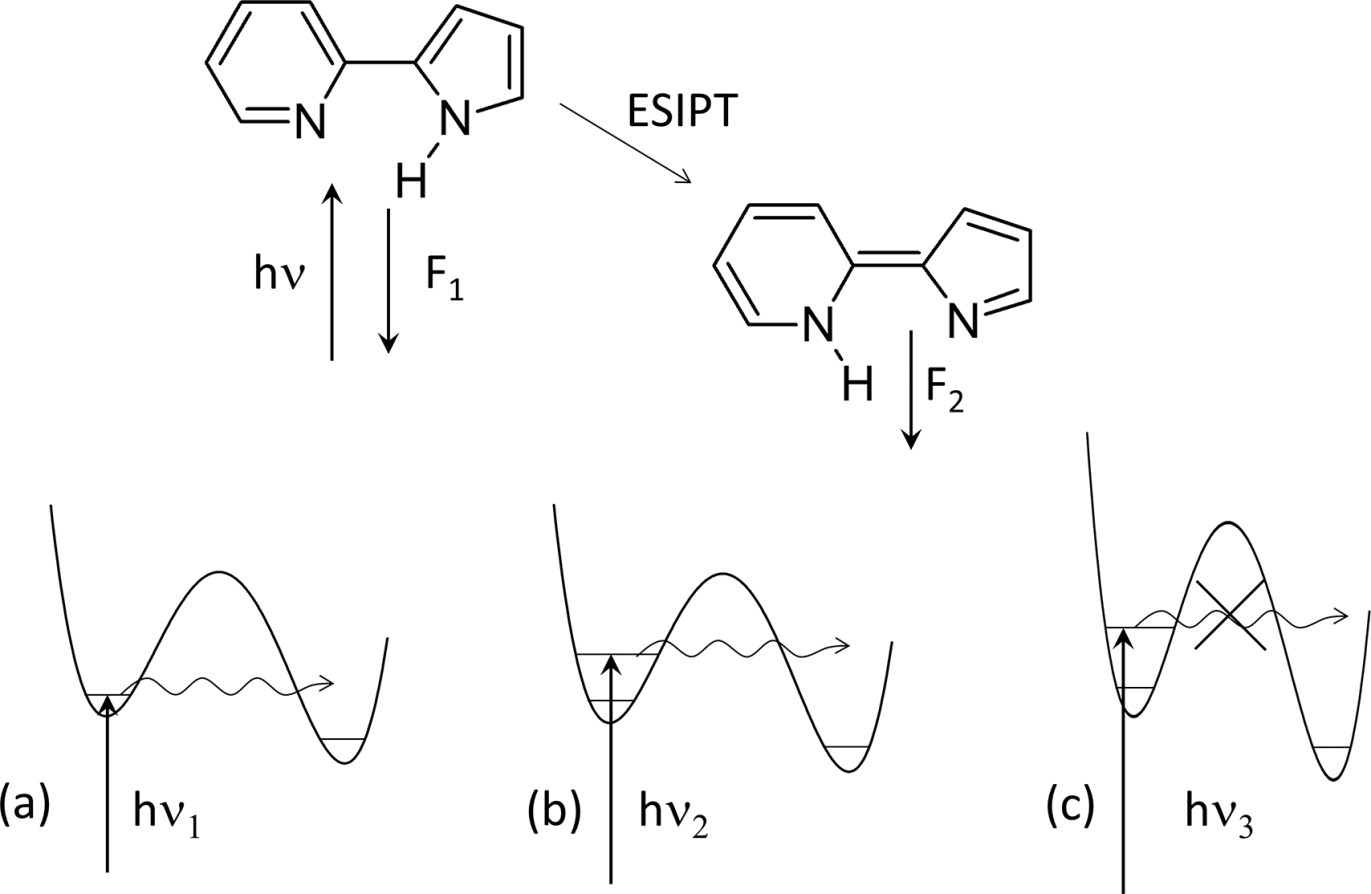
Top: Scheme of excited-state proton transfer (ESIPT) in 2,2′-(pyridyl)pyrrole. F_1_ and F_2_ denote emissions from the initially excited form and the phototautomer, respectively. Bottom: Selective excitation: (a) into the 0–0 band; (b) *v* = 1 level of vibrational mode a; (c) *v* = 1 level of vibrational mode b; *ν*_a_ < *ν*_b_. The photoreaction occurs for cases a and b but not for c. Note that *hν*_1_ < *hν*_2_ < *hν*_3_.

This molecule exhibits dual emission: in addition to “normal” (F_1_) fluorescence from the photoexcited molecule, a weak, strongly red-shifted band (F_2_) is also observed.^5^ It has been assigned to the product of excited-state proton transfer occurring in the lowest excited singlet state (Figure 2). The reaction occurs in a few tens of ps in room-temperature solution, indicating a small barrier. Calculations (CC2, a second-order coupled cluster model) predict a barrier of about 0.2 eV (1610 cm^–1^).^6^

Interestingly, both F_1_ and F_2_ emissions are detected not only in solutions but also in ultracold molecules isolated in supersonic jets. Under these conditions, because of hindered vibrational relaxation, it is possible to compare photoreactivity at different S_1_ vibrational levels (Figure 2, bottom). Analysis of the vibronic structure of the excitation spectra of F_1_ and F_2_ revealed that the formation of the phototautomer is highly excitation wavelength specific.^7^ The tautomer emission is observed upon 0–0 excitation, but when shorter wavelengths are used, only the excitations of two low-frequency vibrational modes, 144 and 352 cm^–1^ (or their overtones or combinations), lead to the photoproduct. The 144 and 352 cm^–1^ bands can be readily assigned to in-plane bending and stretching modes,^8^ for which the atomic displacements strongly modulate the N···N distance and the NH–N angle, i.e., the parameters that characterize the intramolecular hydrogen bond strength.

Large kinetic isotope effects are observed after replacing the NH proton with deuteron. The estimated values are >30 for the 0–0 excitation and >60 for the first overtone of the 144 cm^–1^ band. These results leave no doubt that the phototautomerization in jet-isolated PP occurs via tunneling, either from a zero vibrational level (deep tunneling) or after excitation of specific modes (vibrationally induced tunneling, equivalent to *ta*, the thermally activated process in Figure 1). An important observation is that vibrational excitation may not only promote but also hinder the reaction; the latter is demonstrated by modes that, when excited, stop the generation of the tautomer.

Mode-selectivity can lead to results that seem at first glance counterintuitive: providing more energy to the reactant stops tautomerization, whereas smaller energy quanta promote it. Naturally, what counts is not the amount of energy but the form of the vibrational mode into which the energy is deposited. In addition, the specific excitation must survive for a time period sufficient for the reaction to occur.

### Double H Transfer in Porphyrin

**Figure 3:**
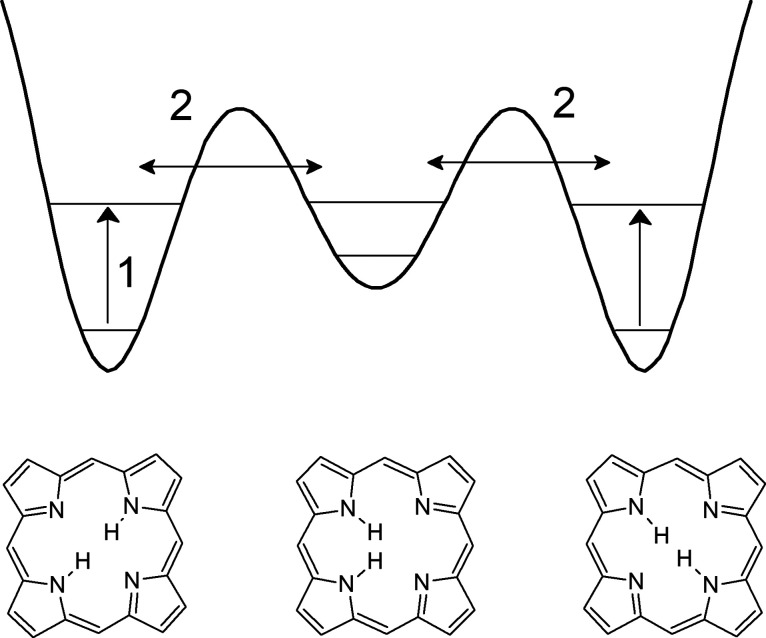
Mechanism of tautomerization in free base porphyrin (porphine): thermal activation (**1**) followed by tunneling (**2**). Note that the *cis* form is quadruply degenerate.

A long-known example of hydrogen transfer controlled by vibrationally induced tunneling is provided by porphyrin (Figure 3). Two degenerate *trans* tautomers interconvert via a stepwise process: the transfer of a single hydrogen atom that leads to a *cis* structure, from which the system can either go back or proceed to the other *trans* form. The analysis of the HH/HD/DD kinetic isotope effect obtained from dynamic NMR experiments, combined with the nonlinear Arrhenius plot, led to the conclusion that the reaction proceeds via thermally activated tunneling.^9^ It was not clear, however, which specific vibrational modes participate in tautomerization: the authors suggested that tunneling can occur from a large number of vibrational states, involving stretching, bending, and skeletal modes.

The activation energies obtained from NMR experiments for solutions of 5,10,15,20-tetraphenylporphyrin (TPP) are quite high: 9.5 kcal/mol for TPP-H_2_, 12.7 kcal/mol for TPP-HD, and 13.2 kcal/mol for TPP-D_2_, indicating a substantial barrier for the reaction.^9^

### Double H Transfer in Porphycene

**Figure 4:**
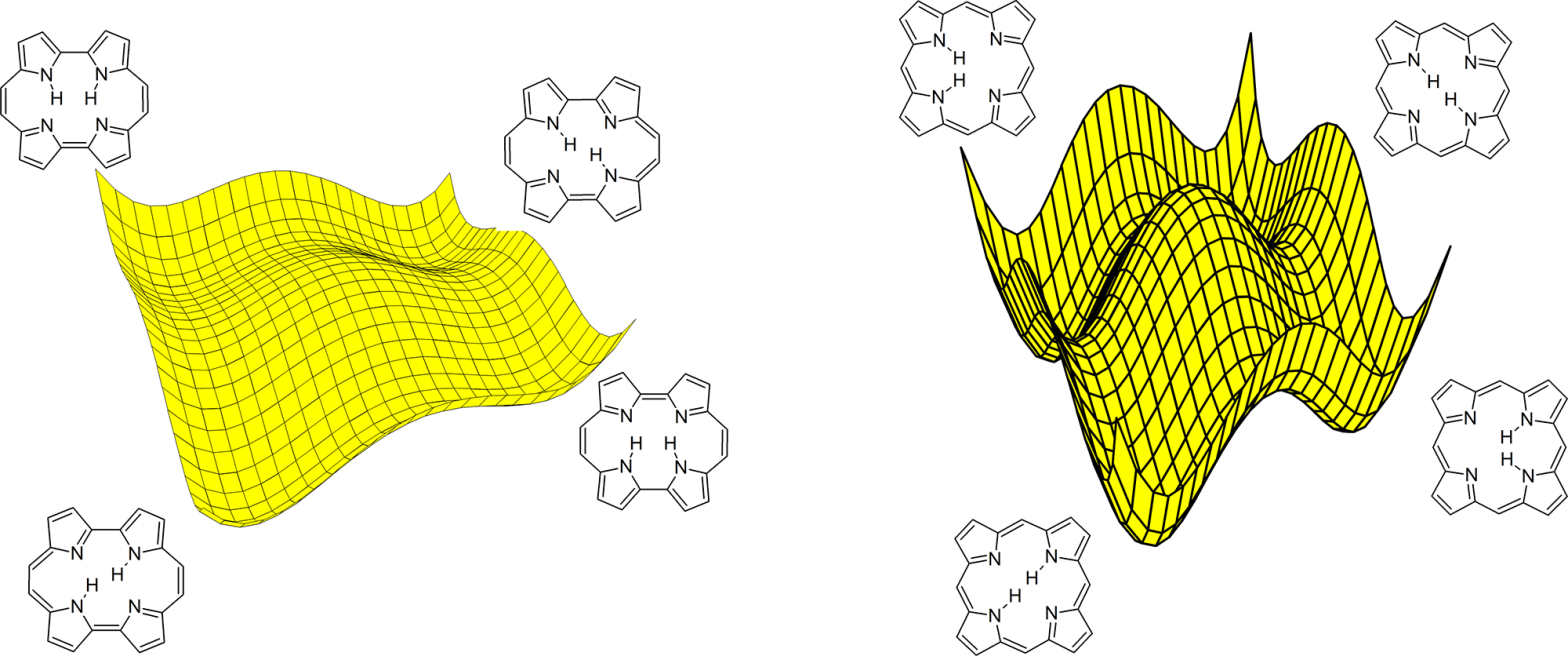
Potential energy surfaces for tautomerization in porphycene (left) and porphyrin (right), drawn on the same scale to visualize the differences in tautomerization barriers.

In room-temperature solutions of TPP-H_2_, tautomerization occurs in several microseconds. Completely different dynamics are encountered in porphycene, a porphyrin isomer (Figure 4). The rectangular shape of the inner cavity results in the intramolecular NH···N H-bonds being much stronger than in porphyrin. The calculated *cis–trans* energy difference is only 2.4 kcal/mol, compared to 8.3 kcal/mol in porphyrin.^10^ Also the barriers for *trans–cis*/*cis–trans* conversions are substantially lower in porphycene: 4.9/2.5 vs 16.2/7.9 kcal/mol in porphyrin.

Since the two *trans* tautomeric forms of porphycene are chemically identical, special techniques were required to measure the rates of the self-exchange *trans–trans* conversion. A procedure was developed for measuring these rates (in both S_0_ and S_1_ electronic states) using polarized pump–probe femtosecond spectroscopy.^11^ In differently substituted porphycenes, tautomerization times measured for room-temperature solutions range from tens of femtoseconds to hundreds of picoseconds.^12^ The rate of double hydrogen transfer is well correlated with structural (N···N distance) and spectral (NH stretching frequency and proton NMR shift) parameters that provide a measure of H-bond strength. The finding that decreasing the N···N separation from 280 pm (2,3,6,7,12,13,16,17-octaethylporphycene) to 253 pm (9,10,19,20-tetramethylporphycene) increases the reaction rate by more than 3 orders of magnitude suggested an important role of tunneling. Another argument was provided by the value of the activation energy obtained for parent porphycene from the linear Arrhenius plot in the temperature range of 90- 293 K. This value, 0.55 kcal/mol, is nearly an order of magnitude lower than the calculated reaction barrier.

**Figure 5:**
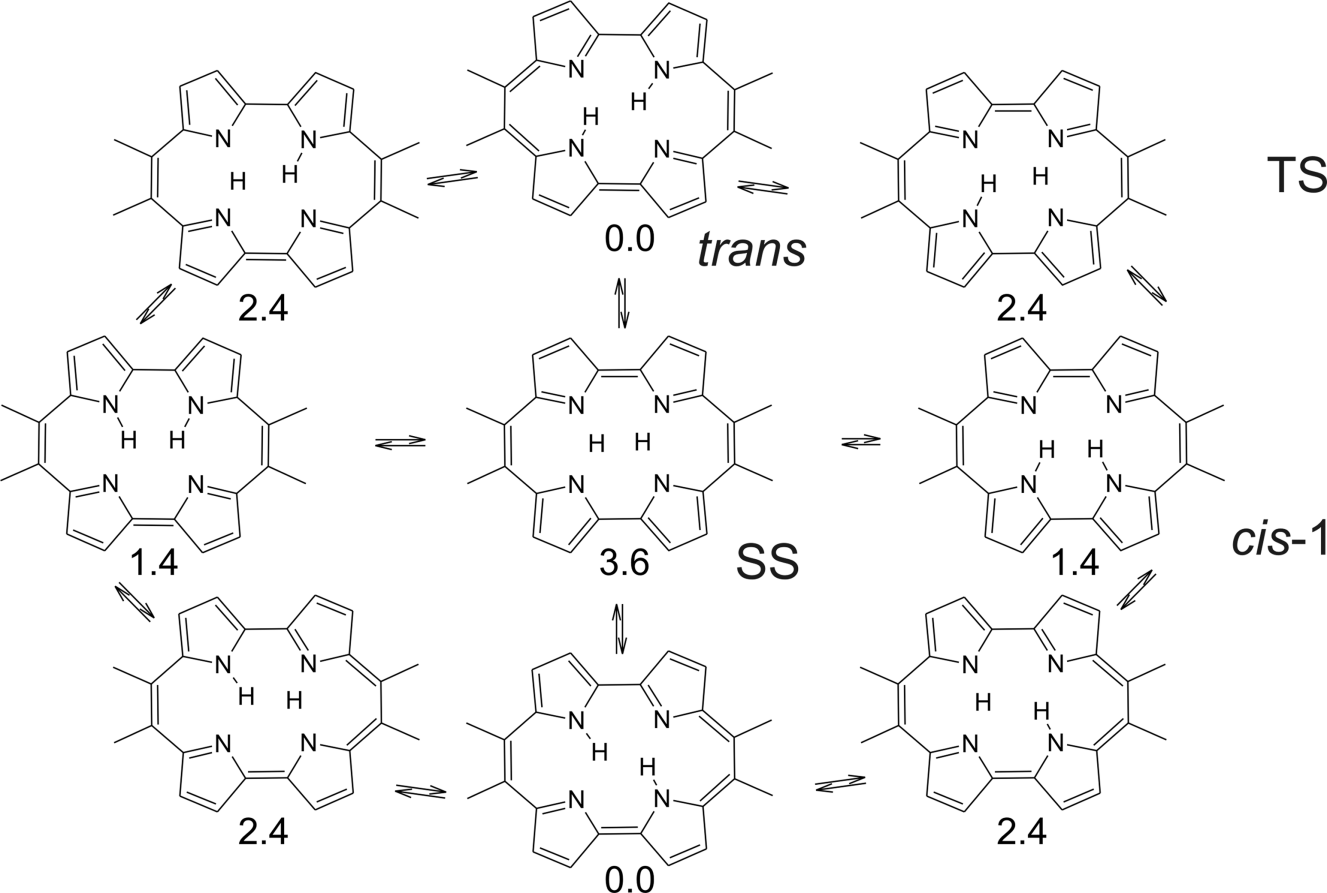
Paths of concerted and stepwise double hydrogen transfer in 9,10,19,20-tetramethylporphycene. The calculated (B3LYP/6-31G(d,p)) energy values (kcal/mol) are given relative to the lowest energy trans form in S_0_. TS, transition state; SS, second-order saddle point.

A spectacular result has been provided by the studies of tautomerization in 9,10,19,20-tetramethylporphycene (Figure 5).^13^ In this molecule, both *trans* and *cis* tautomers are present in a ratio of 4:1 at 293 K. *Trans–trans* conversion is extremely fast: it occurs in 100 fs in S_0_ and in 540 fs in S_1_.^12^ These reaction rates are the largest among all porphycenes investigated so far. The ultrafast rate is consistent with the structural data, which show very small NH···N distances (253 pm), thus indicating very strong H-bonds. However, a quite surprising observation was the biexponential fluorescence decay, implying that *trans* and *cis* forms do not intercovert in S_1_, i.e., on the time scale much longer than that of *trans–trans* conversion. Another unusual feature was a huge dependence of fluorescence lifetimes on solvent viscosity, from a few picoseconds in low viscosity solvents to a few nanoseconds in polymer films. This difference suggested that a large amplitude molecular motion is involved in the radiationless deactivation of S_1_. Calculations suggest that distortion from planarity may occur when one of the internal protons starts moving toward the highest energy (*cis-2*) tautomeric form.^14^

Thermodynamically, stepwise *trans–trans* hydrogen transfer should be favored, as the calculated barrier for the concerted process is higher (Figure 5). However, if the reaction occurs via tunneling, then different paths can be envisaged for the two mechanisms. The calculations suggest that the stepwise process involves coupling with the rotation of methyl substituents, whereas this is not the case in the case of concerted double hydrogen transfer.^13^ As a result, the latter is kinetically preferred.

The above hypotheses about the dominant role of tunneling were confirmed by combining the studies of porphycenes isolated in supersonic jets with experiments performed in condensed phases, from ensemble to single-molecule regimes.

### Tunneling Splittings Observed in Isolated Porphycenes

**Figure 6:**
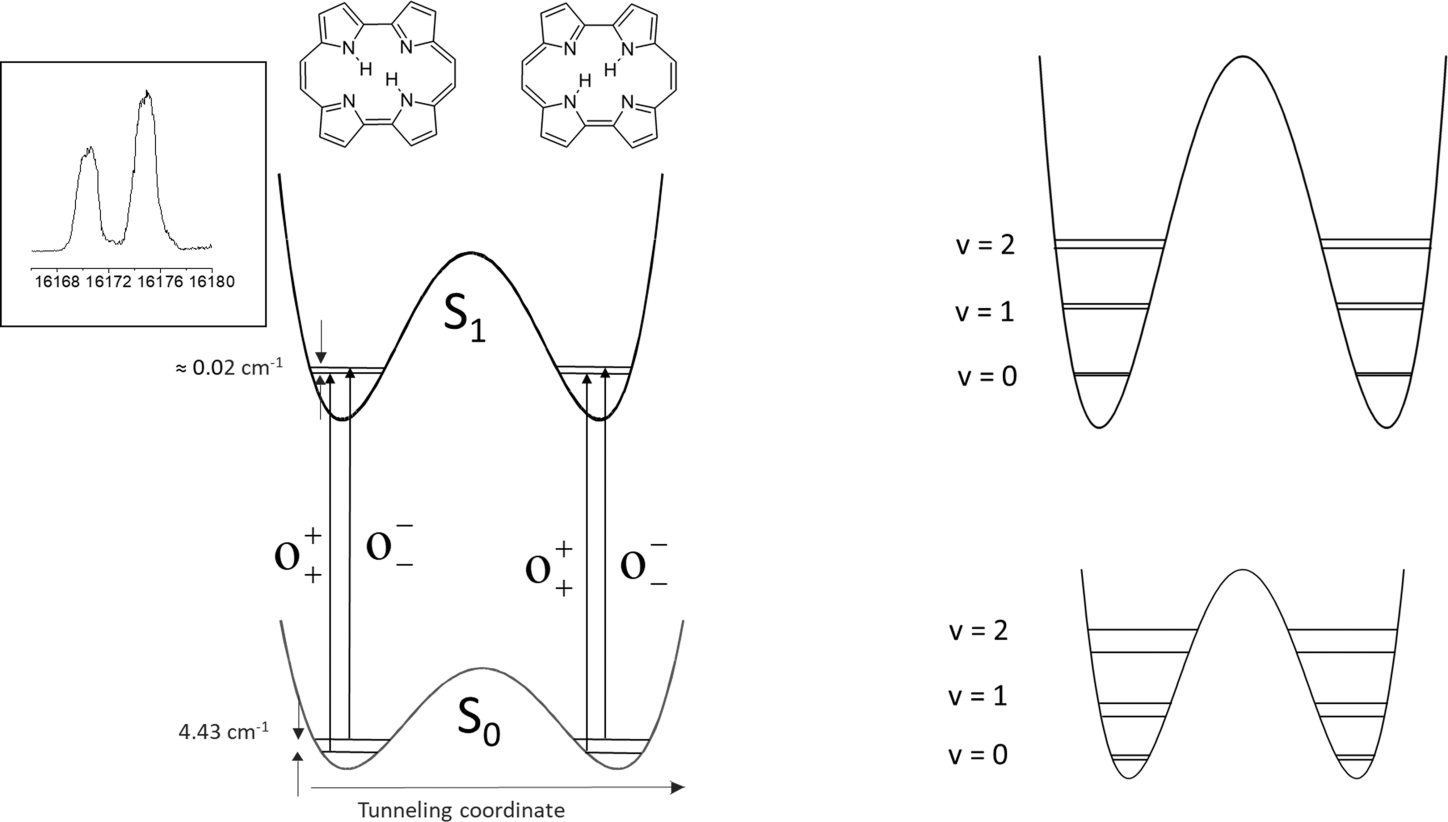
Left: Scheme of tunneling splittings of the vibrational ground states in S_0_ and S_1_. The inset shows the 0–0 doublet in the fluorescence excitation spectrum of porphycene isolated in supersonic jet.^14^ Right: Cross sections of the PES correspond to two vibrational modes that differently affect the strength of the intramolecular H-bonds.

**Figure 7:**
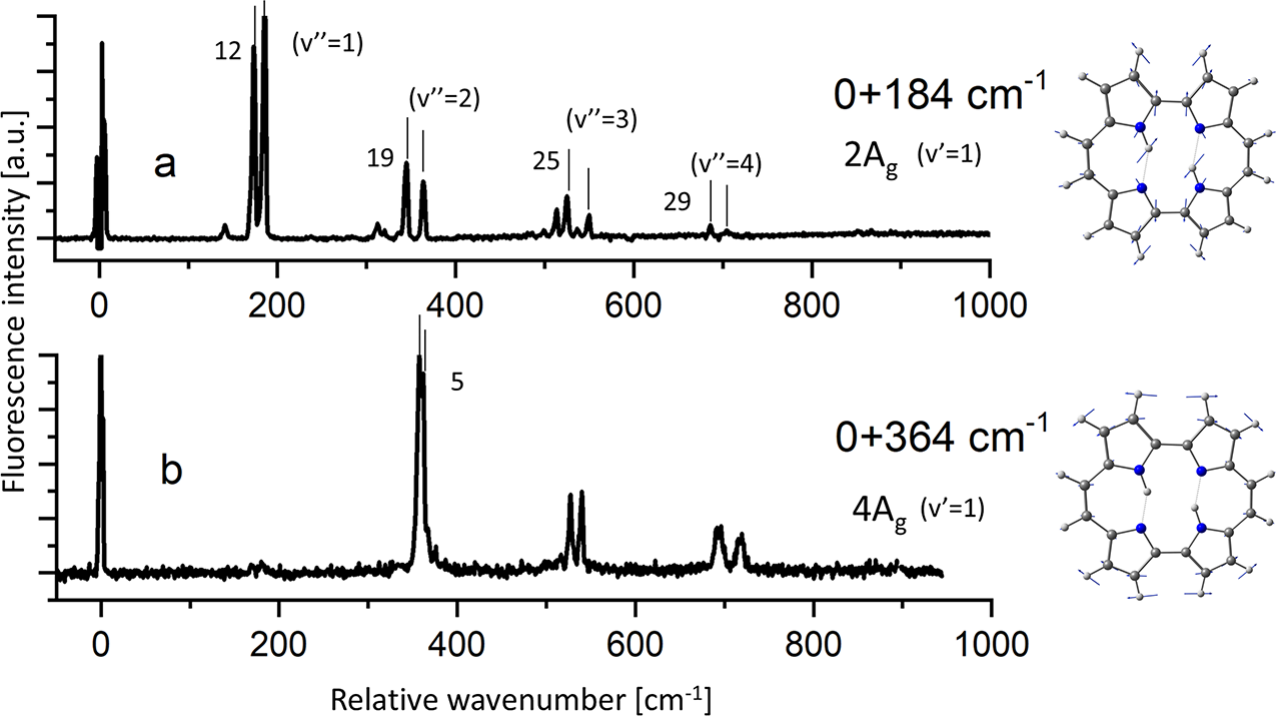
Single vibronic level fluorescence of porphycene in a supersonic jet. Excitation into the *v* = 1 level of the 2A_g_ (a) and 4A_g_ (b) modes. Right, calculated forms of the vibrations.

In an isolated porphycene molecule, the double degeneracy of two *trans* tautomeric forms implies that the two inner protons are coherently delocalized over four nitrogen atoms. This leads to tunneling splittings, which can, in principle, be different for different vibrational and electronic states. This was found to be the case for porphycene (Figures 6 and 7).

Fluorescence and fluorescence excitation spectra of porphycene isolated in supersonic jets^15^ or helium nanodroplets^16^ reveal a doublet structure. It disappears after the replacement of two inner protons by deuterons, proving that the splitting is due to coherent delocalization of the inner protons between the four nitrogen atoms that form the inner cavity. The values of the tunneling splitting are much higher in the ground electronic state than in S_1_: for the vibrationless level, these values are 4.43 cm^–1^ (S_0_) and ∼0.02 cm^–1^ (S_1_) (Figure 6). The difference can be explained by the expansion of the cavity upon electronic excitation. This weakens the two intramolecular H-bonds; as a result, the tautomerization barrier height and width increase.

Since the vibrational excitation can affect the effective distance between the H-bonded atoms (as well as the NH···N angle), one can expect that the values of tunneling splittings may be different for different vibrational modes. Effective shortening of the H-bond and/or making it more linear (smaller barrier height and width) should lead to larger splitting. The opposite is to be expected when the hydrogen bond becomes weaker. Vibrations that strengthen the H-bonds may thus be called “promoting”, whereas those that make them weaker may be classified as “hindering” tautomerization. These two cases are depicted in Figure 7. The third category includes the modes that do not influence the H-bond characteristics: it seems natural to dub them “neutral”.

Observation of fluorescence from single vibronic levels of supersonic-jet isolated porphycene enabled determining the values of the tunneling splittings for different vibrational modes of the ground electronic state (Figure 7).^17^ These values are strongly dependent on the nature of the vibration. For most of the modes, the observed splittings are about 5 cm^–1^, similar to the value determined for the vibrationally unexcited level (see Figure 7b). However, two vibrations revealed very different behavior. For 2A_g_, a large splitting was observed: 12 cm^–1^ for *v* = 1 level, increasing to 29 cm^–1^ for *v* = 4 (Figure 7a). The opposite behavior was observed for 1A_g_, for which the splitting was too small (<1 cm^–1^) to be detected (see the single peak at 150 cm^–1^ in Figure 7a). The two modes thus provide examples of promoting (2A_g_) and hindering (1A_g_) vibrations, whereas the 4A_g_ mode can be classified as neutral.

The calculated forms of the 2A_g_ and 4A_g_ modes (Figure 7) allow for an intuitive understanding of the difference in tunneling splitting values. During the 2A_g_ vibration, both H-bonds become shorter and more linear. In contrast, the cavity atoms practically do not move in 4A_g_. One should expect a larger splitting in the former, whereas for the latter, a value similar to that of the vibrationally unexcited state is not surprising. Interestingly, this simple picture was nicely confirmed in another supersonic jet study. Porphycene-*d*_12_, an isotopologue with all peripheral protons substituted by deuterons, revealed for 4A_g_ a splitting of 9 cm^–1^, twice that as observed for the vibrationless level.^18^ Displacement vectors calculated for the 4A_g_ mode in porphycene-*d*_12_ showed the movement of the inner atoms in the deuterated derivative. In other words, deuteration at the periphery changed the character of the 4A_g_ mode from neutral into promoting.

Supersonic jet studies have also been performed for 9,10,19,20-tetramethylporphycene.^19^ The tunneling splittings observed for this molecule were larger than those for the unsubstituted porphycene, as could have been expected based on weaker H-bonds in the latter. These studies also confirmed the hypothesis of coupling between hydrogen transfer and rotation of the methyl groups. These studies can be considered an extension of earlier investigations of hydroxyphenalenones^20^ and tropolones^21^ which discussed how tunneling is affected by the coupling of methyl group rotation with a single proton transfer.

## Environmental Effects

### Double Hydrogen Tunneling in Condensed Phases

One can wonder whether the results obtained for cold, isolated molecules that exhibit coherent delocalization of the inner hydrogens may have relevance to the mechanism of tautomerization in condensed phases. First, one can ask whether tunneling is important at all at elevated temperatures. Second, even if it is, one expects a noncoherent process caused by interaction with the environment, which temporarily stabilizes one of the tautomeric forms.

**Figure 8:**
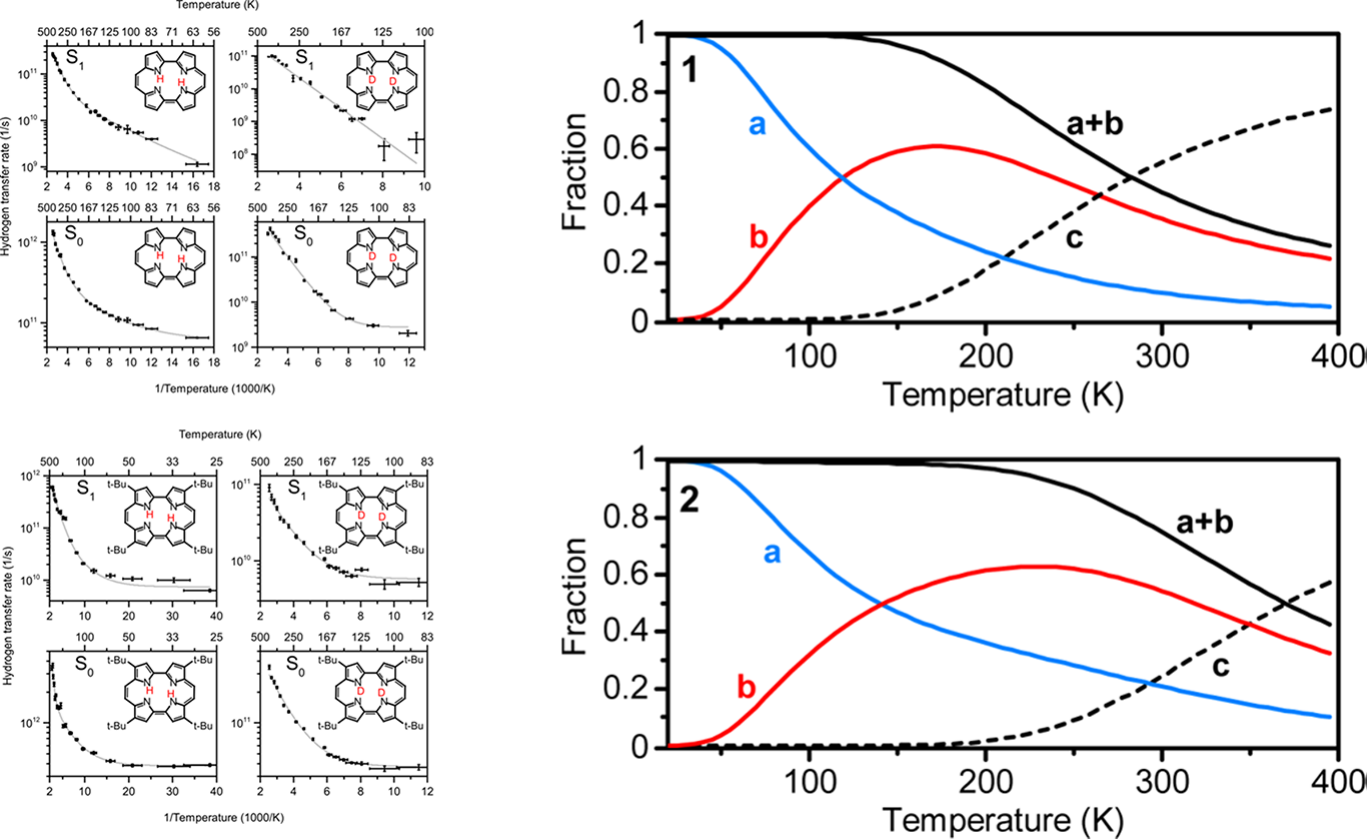
Left: Double hydrogen transfer rates determined for nondeuterated and NH-deuterated porphycene (1) and 2,7,12,17-tetra-*t*-butylporphycene (2) in the electronic ground (S_0_) and lowest excited singlet (S_1_) states. Adapted from ref (22). Copyright 2016 American Chemical Society. Right: Contribution of three reaction channels to the overall rate in the ground state at different temperatures. Adapted from ref (22). Copyright 2016 American Chemical Society.

Using polarized pump–probe spectroscopy, the rates of tautomerization have been measured over a wide temperature range (20–400 K) for two porphycenes and their N-deuterated analogues.^22^ The obtained values (Figure 8) could be well fitted by a model assuming that the rate can be expressed by the equation
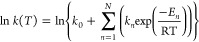
where *N* = 2. In other words, three channels contribute to the reaction rate. The first term (*k*_0_) is temperature-independent; it can be attributed to tunneling from the vibrational ground state. The activation energy found for the first temperature-dependent channels is 0.5 kcal/mol. It is equal, within experimental error, to 180 cm^–1^, the frequency of the 2A_g_ mode that exhibits the largest tunneling splitting in isolated porphycene (as determined for both undeuterated and singly N-deuterated molecule^16^). Therefore, the second channel was assigned to vibrationally induced tunneling. For *E*_2_, the values of 1.5–3.7 kcal/mol were determined; they were somewhat different for the two molecules.

The plot showing relative contributions of the three channels to the total reaction rate (Figure 8) clearly shows that channels (a) + (b), corresponding to tunneling (deep and vibrationally activated, respectively), are dominant even at room temperature. The nature of the third channel is less clear. It can be assigned to either over-the-barrier crossing or to excitation of another promoting mode. The latter seems more probable, because the activation energy found for this channel is still much lower than the calculated barrier. A mode at 947 cm^–1^ (2.7 kcal/mol), calculated for porphycene, has been proposed as the candidate for *trans–cis* conversion, because the displacement vectors show that while one of the H-bonds contracts, the other becomes elongated.^23^ It is therefore possible that channels (b) and (c) represent concerted and stepwise double hydrogen transfers, respectively.

The values of the reaction rates obtained from studies in condensed phases were compatible with the data found for jet-isolated molecules.^22^ The residence time in the coherent process (τ = *h*/(2Δ), where Δ is the observed splitting) was shorter than the inverse of *k*_0_, which could be expected because of solvent-induced perturbation of the symmetric double minimum potential, making the reaction slower. In turn, for the incoherent tunneling regime, the rate is proportional to Δ^2^. Excellent agreement was found: *k*_1_/*k*_0_ = 7.0, to be compared with (12/4.4)^2^ = 7.4, the value obtained from the ratio of the squares of tunneling splittings in the vibrational ground state and the *v* = 1 level of the 2A_g_ mode.

One observation, however, was not easy to rationalize. The contribution from channel b, i.e., activation of tunneling by excitation of the 2A_g_ mode decreased significantly in the deuterated porphycenes (where the 2A_g_ frequency remains the same), whereas one could have expected the opposite, given a larger barrier due to lowering of zero-point energy. Various explanations can be considered. Based on calculations, the change of the character of 2A_g_, as discussed above for 4A_g_, seems improbable. Another possibility is the periodic perturbation of the symmetric double minimum potential by the environment, which may strongly affect the tunneling probability when the mass becomes larger.^24^ Finally, we note that the double hydrogen transfer in nondeuterated porphycenes **1** and **2** occurs in a picosecond or even less, whereas deuteration slows this process by nearly an order of magnitude. The lifetime of the *v* = 1 2A_g_ vibrational level can be reasonably estimated, based on experiments using nondeuterated porphycene, as ca. 10 ps;^25^ it may therefore be sufficiently long to maintain the mode selectivity in parent **1** or **2**, but not in their -*d*_2_ isotopologues. Anyway, this intriguing observation calls for further studies.

A word of comment seems appropriate regarding the assumed noncoherent nature of the hydrogen transfer in the condensed phase. For porphycene in acetonitrile at 293 K, the evolution of wavepackets in both S_0_ and S_1_ states lasts about 2 ps, which is very close to the tautomerization time. One can consider that what the experiment using ultrashort laser excitation is probing is not exactly a noncoherent process.

### Tunneling in Single Molecules

**Figure 9:**
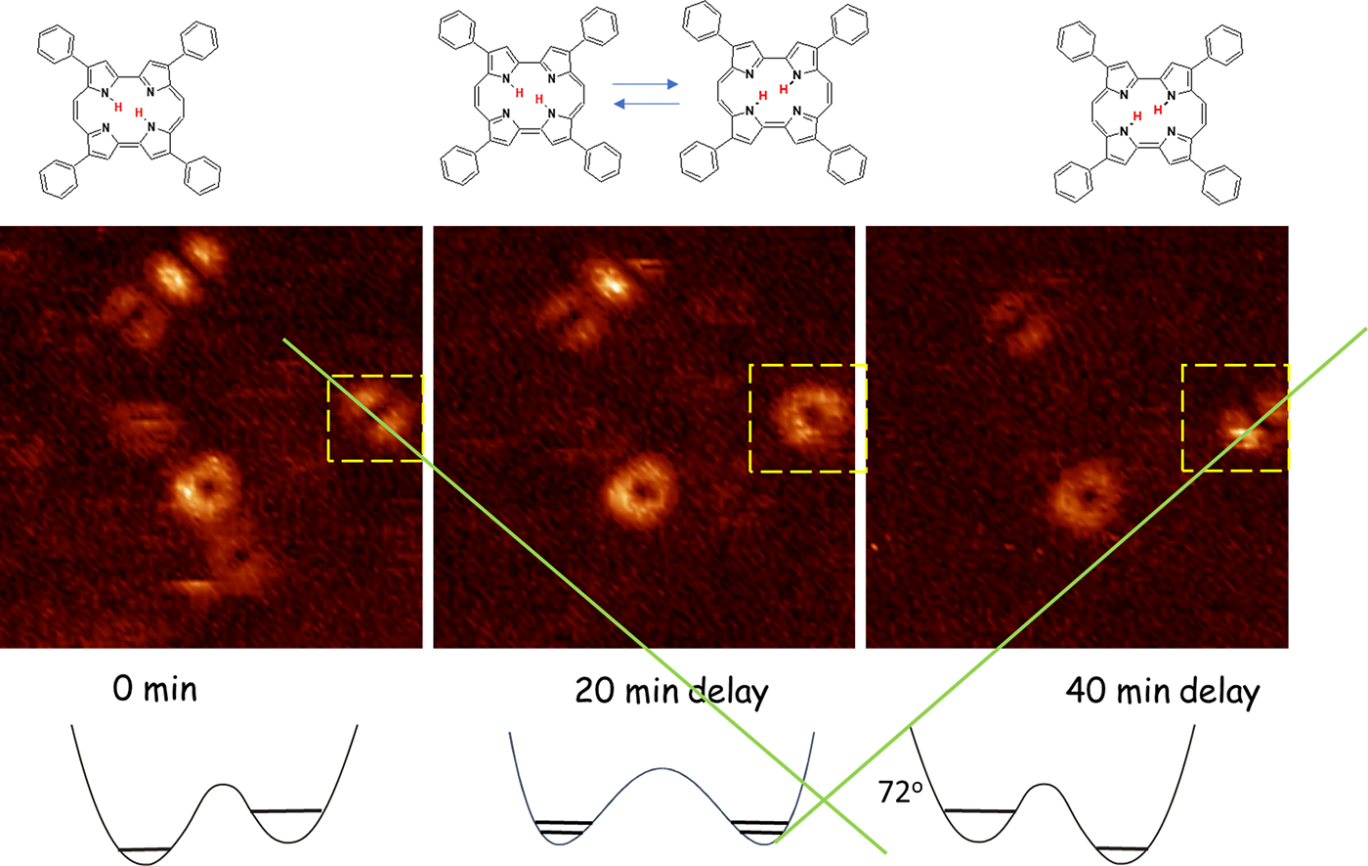
Confocal fluorescence images of the emission from single molecules of 2,7,12,17-tetraphenylporphycene at 293 K. Left to right: three consecutive scans taken at 20 min intervals. The molecule labeled by a yellow square switches between a regime of localized protons (double lobe shape) and that of the molecule undergoing rapid *trans*–*trans* conversion (doughnut shape). The angle between the symmetry axes of the double lobes (green lines), 72°, is identical to the angle formed by S_1_ ← S_0_ transition in the two trans tautomers.^25^ Bottom: schematic representation of the potential energy curve along the reaction path during each scan.

Porphycene is one of very few molecules (if not the only one!) that have been investigated on a single-molecule level using three different techniques: confocal fluorescence and Raman as well as scanning probe microscopy. Using an appropriately polarized laser beam for excitation, it was possible to observe *trans–trans* double hydrogen transfer by looking at the shape of the confocal single-molecule fluorescence image.^26^ These experiments led to an interesting finding: in molecules embedded in a polymer film, the tautomerization rate was changing over time.^27,28^ The image of a single molecule was evolving over minutes between (i) that of localized protons (double lobe shape), (ii) that of a rapidly tautomerizing molecule (ring shape), and (iii) that of protons localized on two nitrogens other than in (i) (Figure 9). The ensemble studies of the same molecules in room-temperature solutions revealed tautomerization times of a picosecond, or even less.^12^ No significant solvent dependence was found. On the other hand, experiments carried out for polymer samples containing porphycene revealed at low temperatures the presence of two populations; in the first one, tautomerization was occurring on the subnanosecond time scale, whereas in the other it was too slow to be detected by nanosecond techniques.^29^ In poly(methyl methacrylate) (PMMA), the fraction of hydrogen-transferring molecules was found to obey an Arrhenius-like equation, with the activation energy of 68 ± 2 cm^–1^. This value coincides with 70 cm^–1^, a low-frequency mode of PMMA.

The above results fit nicely to the model of the double minimum potential being modulated by the slow motions of the polymer matrix, which hinders tunneling. The reaction rate is controlled by the environment relaxation, and the transfer occurs when the potential becomes symmetric. The huge difference in the rates observed in solutions and polymers is due to the different time scales of the solvent relaxation dynamics. In the case of polymer matrices, their relaxation must be explicitly included in the reaction path.

**Scheme 1:**
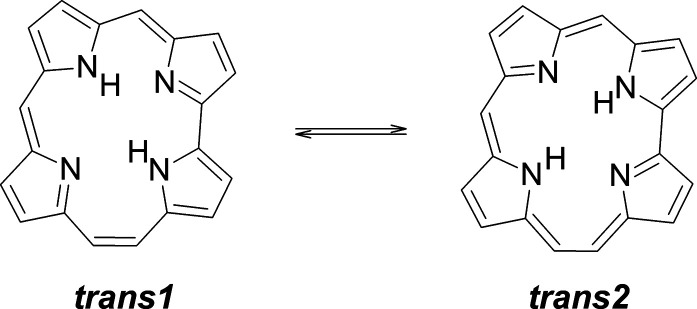
Chemically Nonequivalent *trans* Tautomers of Hemiporphycene

Analogous results have been obtained in single-molecule studies of tautomerization in hemiporphycene, another porphyrin isomer (Scheme 1).^30^ In this case, the two *trans* tautomers are not equivalent, so their electronic absorption and emission spectra differ. Analysis of the confocal fluorescence spectra from single hemiporphycene molecules revealed that the *trans*–*trans* conversion can be very slow, indicating that the reaction dynamics is controlled, as in porphycenes, by the polymer relaxation. It should be noted, though, that, contrary to the situation in porphycenes, the hydrogen tunneling can now be, in principle, not necessarily slowed down but also accelerated by the matrix motions, since the potential, nonsymmetric in the isolated molecule, could become more symmetric as a result of interaction with the polymer environment.

Studies of tautomerization in single porphycene molecules were also carried out using scanning probe microscopy in the regime of high vacuum and cryogenic temperatures. The molecules were deposited on the surface of a metal. Under these conditions, for certain crystal surfaces (e.g., Cu(110)^31^ or Ag(110)^32^) the *cis* form is stabilized with respect to the *trans* species, so that it becomes the lowest energy tautomer. At 5 K, tautomerization can proceed only via tunneling. This process was found to occur for porphycene on Ag(110), but not on Cu(110). Analysis of kinetic isotope effects observed for porphycene and its singly and doubly N-deuterated derivatives placed on Ag(110) demonstrated that the *cis–cis* conversion occurred as a stepwise process. Interestingly, the tautomerization coordinate now involves the whole molecule, as the *cis–cis* conversion is accompanied by displacements of nitrogen and carbon atoms. As expected, below 10 K, the reaction rate is temperature-independent, exhibiting an isotope ratio of about 100 between porphycene-*d*_0_ and porphycene-*d*_2_.

**Figure 10:**
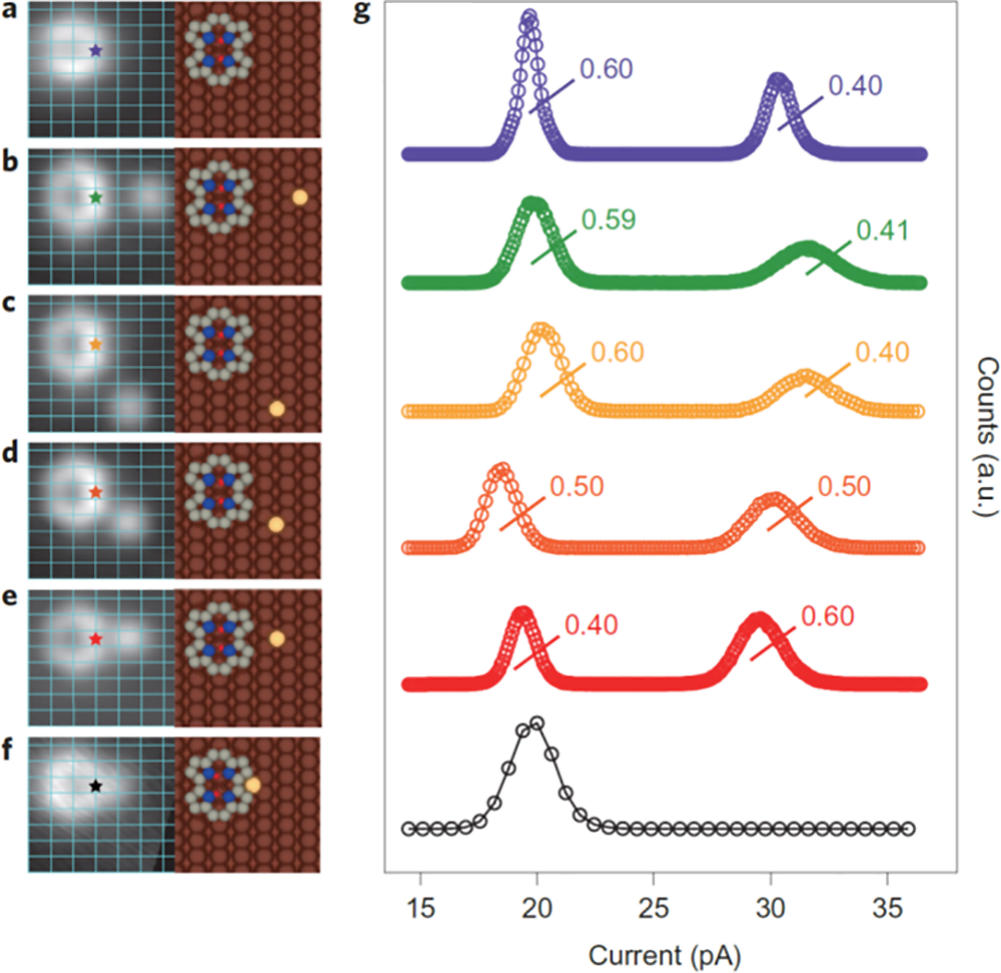
Left: STM images of a single porphycene molecule lying on a Cu(110) surface at 5 K, without (a) or with a single Cu adatom (b–f) placed nearby. Right: Current histograms showing changes in the relative populations of two *cis* tautomers. Adapted from ref (31).

In the case of porphycene on Cu(110), the reaction could still be induced by external factors, such as vibrational excitation by STM electrons,^33^ placing a single atom close to the molecule (Figure 10),^31^ light,^34^ or mechanical interaction with the tip.^35^

The STM experiments highlight the extreme sensitivity of tunneling to the environment. The possibility of affecting the reaction path by external stimuli provides an attractive perspective of practical use of porphycene and analogous systems, e.g., as single-molecule switches or memories.

In summary, the experimental results obtained for the systems presented above clearly indicate the crucial role of tunneling in the ground state and in photoinduced tautomerization. For 2,2′-(pyridyl)pyrrole, it remains to be established whether this mechanism is also dominant at room temperature, as was found to be the case for porphyrin and its isomers. The results obtained for structurally similar compounds^36,37^ strongly suggest such a possibility.

While tunneling is clearly a favorable option for systems with high reaction barrier, such as porphyrin, its dominance at normal temperatures for porphycenes, characterized by low or very low barriers, is not so obvious. In this respect, it could be instructive to assess the contribution from tunneling for several tautomerizing molecules with similar barriers but various structures, in particular regarding the possibility of modulating the H-bond strength by vibrations. Recent works on this subject focus on molecules topologically similar to the ones discussed in this work, but chemically somewhat different: tropolone, a paradigm of proton tunneling in a symmetrical double well potential,^38^ and a newly discovered system: 6-hydroxy-2-formylfulvene.^39,40^ Both represent OH···O proton transfer models.

The fascinating feature of vibrationally induced tunneling is its mode selectivity. Gaining control over this phenomenon under “normal” conditions would lead to the realization of mode-specific chemistry or photochemistry, a goal pursued for many years. In order to achieve that, tight cooperation between theoreticians and experimentalists is absolutely necessary. Regarding the latter, development of ultrafast IR and Raman techniques that can probe the dynamics and coupling of vibrational modes during the reaction is extremely promising. An example is provided by investigation of ground and excited proton transfer dynamics in green fluorescence protein (GFP), suggesting not only tunneling but also coherent nuclear motions.^41^ GFP has been proposed as an excellent model system for studying the fundamental reaction mechanisms. In our opinion, somewhat simpler systems, like the molecules presented in this work, seem more realistic (although “simpler” in these cases still looks quite complex!). In fact, tautomerization in porphycene has been already quite intensely studied by theory, using various approaches.^42,43^ The calculations not only nicely reproduced the reaction rates but also indicated a competition between stepwise and concerted hydrogen transfer dynamics, suggested by experiment.

Finally, we note that the difference in the effective tautomerization barrier perceived by a molecule excited to different vibrational states calls for an extension of the definition of the hydrogen bond beyond that based on thermodynamics. Otherwise, it would be difficult to explain why the rate of tautomerization, strongly related to the H-bond strength, can either increase or decrease upon pumping energy into a molecule.

## References

[ref1] Hund F. Zur Deutung Der Molekelspektren. III. Z. Phys. 1927, 43, 805–826. 10.1007/BF01397249.

[ref2] Schleif T.; Prado Merini M.; Henkel S.; Sander W. Solvation Effects on Quantum Tunneling Reactions. Acc. Chem. Res. 2022, 55, 2180–2190. 10.1021/acs.accounts.2c00151. 35730754

[ref3] Borden W. T. Reactions That Involve Tunneling by Carbon and the Role That Calculations Have Played in Their Study. WIREs Comput. Mol. Sci. 2016, 6, 20–46. 10.1002/wcms.1235.

[ref4] Schäfer M.; Peckelsen K.; Paul M.; Martens J.; Oomens J.; Berden G.; Berkessel A.; Meijer A. J. H. M. Hydrogen Tunneling above Room Temperature Evidenced by Infrared Ion Spectroscopy. J. Am. Chem. Soc. 2017, 139, 5779–5786. 10.1021/jacs.6b10348. 28282985

[ref5] Kijak M.; Zielińska A.; Chamchoumis C.; Herbich J.; Thummel R. P.; Waluk J. Conformational Equilibria and Photoinduced Tautomerization in 2-(2′-Pyridyl)Pyrrole. Chem. Phys. Lett. 2004, 400, 279–285. 10.1016/j.cplett.2004.10.121.

[ref6] Rode M. F.; Sobolewski A. L. Photophysics of Inter- and Intra-Molecularly Hydrogen Bonded Systems:: Computational Studies on the Pyrrole-Pyridine Complex and 2-(2′-Pyridyl)Pyrrole. Chem. Phys. 2008, 347, 413–421. 10.1016/j.chemphys.2007.11.013.

[ref7] Kijak M.; Nosenko E.; Singh A.; Thummel R. P.; Waluk J. Mode-Selective Excited State Proton Transfer in 2-(2′-Pyridyl)Pyrrole Isolated in a Supersonic Jet. J. Am. Chem. Soc. 2007, 129, 2738–2739. 10.1021/ja068109f. 17305339

[ref8] Kijak M.; Nosenko Y.; Singh A.; Thummel R. P.; Brutschy B.; Waluk J. Ground and Excited State Vibrations of 2-(2′-Pyridyl)Indole. J. Mol. Struct. 2007, 844-845, 286–299. 10.1016/j.molstruc.2007.05.019.

[ref9] Schlabach M.; Wehrle B.; Rumpel H.; Braun J.; Scherer G.; Limbach H. H. NMR and NIR Studies of the Tautomerism of 5,10,15,20-Tetraphenylporphyrin, Including Kinetic HH/HD/DD Isotope and Solid State Effects. Ber. Bunsen-Ges. Phys. Chem. 1992, 96, 821–833. 10.1002/bbpc.19920960616.

[ref10] Kozlowski P. M.; Zgierski M. Z.; Baker J. The Inner-Hydrogen Migration and Ground-State Structure of Porphycene. J. Chem. Phys. 1998, 109, 5905–5913. 10.1063/1.477213.

[ref11] Fita P.; Urbańska N.; Radzewicz C.; Waluk J. Ground and Excited State Tautomerization Rates in Porphycenes. Chem.- Eur. J. 2009, 15, 4851–4856. 10.1002/chem.200802428. 19308981

[ref12] Ciąćka P.; Fita P.; Listkowski A.; Kijak M.; Nonell S.; Kuzuhara D.; Yamada H.; Radzewicz C.; Waluk J. Tautomerism in Porphycenes: Analysis of Rate-Affecting Factors. J. Phys. Chem. B 2015, 119, 2292–2301. 10.1021/jp506150r. 25105931

[ref13] Gil M.; Dobkowski J.; Wiosna-Sałyga G.; Urbańska N.; Fita P.; Radzewicz C.; Pietraszkiewicz M.; Borowicz P.; Marks D.; Glasbeek M.; Waluk J. Unusual, Solvent Viscosity-Controlled Tautomerism and Photophysics: *Meso*-Alkylated Porphycenes. J. Am. Chem. Soc. 2010, 132, 13472–13485. 10.1021/ja105353m. 20825186

[ref14] Kijak M.; Nawara K.; Listkowski A.; Masiera N.; Buczyńska J.; Urbańska N.; Orzanowska G.; Pietraszkiewicz M.; Waluk J. 2 + 2 Can Make Nearly a Thousand! Comparison of Di- and Tetra-Meso-Alkyl-Substituted Porphycenes. J. Phys. Chem. A 2020, 124, 4594–4604. 10.1021/acs.jpca.0c02155. 32423205 PMC7590974

[ref15] Sepioł J.; Stepanenko Y.; Vdovin A.; Mordziński A.; Vogel E.; Waluk J. Proton Tunnelling in Porphycene Seeded in a Supersonic Jet. Chem. Phys. Lett. 1998, 296, 549–556. 10.1016/S0009-2614(98)01085-9.

[ref16] Vdovin A.; Waluk J.; Dick B.; Slenczka A. Mode-Selective Promotion and Isotope Effects of Concerted Double-Hydrogen Tunneling in Porphycene Embedded in Superfluid Helium Nanodroplets. ChemPhysChem 2009, 10, 761–765. 10.1002/cphc.200900022. 19229893

[ref17] Mengesha E. T.; Sepioł J.; Borowicz P.; Waluk J. Vibrations of Porphycene in the S_0_ and S_1_ Electronic States: Single Vibronic Level Dispersed Fluorescence Study in a Supersonic Jet. J. Chem. Phys. 2013, 138, 174201 10.1063/1.4802769. 23656125

[ref18] Mengesha E. T.; Zehnacker-Rentien A.; Sepioł J.; Kijak M.; Waluk J. Spectroscopic Study of Jet-Cooled Deuterated Porphycenes: Unusual Isotopic Effects on Proton Tunneling. J. Phys. Chem. B 2015, 119, 2193–2203. 10.1021/jp505553z. 25137228

[ref19] Vdovin A.; Sepioł J.; Urbańska N.; Pietraszkiewicz M.; Mordziński A.; Waluk J. Evidence for Two Forms, Double Hydrogen Tunneling, and Proximity of Excited States in Bridge-Substituted Porphycenes: Supersonic Jet Studies. J. Am. Chem. Soc. 2006, 128, 2577–2586. 10.1021/ja054745m. 16492041

[ref20] Nishi K.; Sekiya H.; Mochida T.; Sugawara T.; Nishimura Y. Coupling between the Internal Rotation of the Methyl Group and Proton/Deuteron Transfer in Jet-Cooled 5-Methyl-9-Hydroxyphenalenone(OH) and 5-Methyl-9-Hydroxyphenalenone(OD): Tunneling Rate Dependence of Coupling Potential. J. Chem. Phys. 2000, 112, 5002–5011. 10.1063/1.481055.

[ref21] Nishi K.; Sekiya H.; Kawakami H.; Mori A.; Nishimura Y. Tunneling in Jet-Cooled 5-Methyltropolone and 5-Methyltropolone–Od. Coupling between Internal Rotation of Methyl Group and Proton Transfer. J. Chem. Phys. 1999, 111, 3961–3969. 10.1063/1.479698.

[ref22] Ciąćka P.; Fita P.; Listkowski A.; Radzewicz C.; Waluk J. Evidence for Dominant Role of Tunneling in Condensed Phases and at High Temperatures: Double Hydrogen Transfer in Porphycenes. J. Phys. Chem. Lett. 2016, 7, 283–288. 10.1021/acs.jpclett.5b02482. 26727277

[ref23] Shibl M. F.; Pietrzak M.; Limbach H. H.; Kühn O. Geometric H/D Isotope Effects and Cooperativity of the Hydrogen Bonds in Porphycene. ChemPhysChem 2007, 8, 315–321. 10.1002/cphc.200600511. 17177226

[ref24] Lignier H.; Sias C.; Ciampini D.; Singh Y.; Zenesini A.; Morsch O.; Arimondo E. Dynamical Control of Matter-Wave Tunneling in Periodic Potentials. Phys. Rev. Lett. 2007, 99, 220403 10.1103/PhysRevLett.99.220403. 18233266

[ref25] Fita P.; Radzewicz C.; Waluk J. Electronic and Vibrational Relaxation of Porphycene in Solution. J. Phys. Chem. A 2008, 112, 10753–10757. 10.1021/jp8049697. 18828574

[ref26] Piwoński H.; Stupperich C.; Hartschuh A.; Sepioł J.; Meixner A.; Waluk J. Imaging of Tautomerism in a Single Molecule. J. Am. Chem. Soc. 2005, 127, 5302–5303. 10.1021/ja043265c. 15826151

[ref27] Piwoński H.; Sokołowski A.; Kijak M.; Nonell S.; Waluk J. Arresting Tautomerization in a Single Molecule by the Surrounding Polymer: 2,7,12,17-Tetraphenylporphycene. J. Phys. Chem. Lett. 2013, 4, 3967–3971. 10.1021/jz4022602.

[ref28] Piatkowski L.; Schanbacher C.; Wackenhut F.; Jamrozik A.; Meixner A. J.; Waluk J. Nature of Large Temporal Fluctuations of Hydrogen Transfer Rates in Single Molecules. J. Phys. Chem. Lett. 2018, 9, 1211–1215. 10.1021/acs.jpclett.8b00299. 29470087

[ref29] Kasprzycki P.; Kopycki P.; Listkowski A.; Gorski A.; Radzewicz C.; Birch D. J. S.; Waluk J.; Fita P. Influence of Local Microenvironment on the Double Hydrogen Transfer in Porphycene. Phys. Chem. Chem. Phys. 2020, 22, 17117–17128. 10.1039/D0CP02687E. 32687131

[ref30] Kim V.; Piatkowski L.; Pszona M.; Jäger R.; Ostapko J.; Sepioł J.; Meixner A. J.; Waluk J. Unusual Effects in Single Molecule Tautomerization: Hemiporphycene. Phys. Chem. Chem. Phys. 2018, 20, 26591–26596. 10.1039/C8CP05836A. 30310894

[ref31] Kumagai T.; Hanke F.; Gawinkowski S.; Sharp J.; Kotsis K.; Waluk J.; Persson M.; Grill L. Controlling Intramolecular Hydrogen Transfer in a Porphycene Molecule with Single Atoms or Molecules Located Nearby. Nat. Chem. 2014, 6, 41–46. 10.1038/nchem.1804. 24345945

[ref32] Koch M.; Pagan M.; Persson M.; Gawinkowski S.; Waluk J.; Kumagai T. Direct Observation of Double Hydrogen Transfer Via Quantum Tunneling in a Single Porphycene Molecule on a Ag(110) Surface. J. Am. Chem. Soc. 2017, 139, 12681–12687. 10.1021/jacs.7b06905. 28826219

[ref33] Kumagai T.; Hanke F.; Gawinkowski S.; Sharp J.; Kotsis K.; Waluk J.; Persson M.; Grill L. Thermally and Vibrationally Induced Tautomerization of Single Porphycene Molecules on a Cu(110) Surface. Phys. Rev. Lett. 2013, 111, 246101–5. 10.1103/PhysRevLett.111.246101. 24483678

[ref34] Böckmann H.; Liu S.; Mielke J.; Gawinkowski S.; Waluk J.; Grill L.; Wolf M.; Kumagai T. Direct Observation of Photoinduced Tautomerization in Single Molecules at a Metal Surface. Nano Lett. 2016, 16, 1034–1041. 10.1021/acs.nanolett.5b04092. 26796945

[ref35] Ladenthin J.; Frederiksen T.; Persson M.; Sharp J.; Gawinkowski S.; Waluk J.; Kumagai T. Force-Induced Tautomerization in a Single Molecule. Nat. Chem. 2016, 8, 935–940. 10.1038/nchem.2552. 27657869

[ref36] Marks D.; Zhang H.; Borowicz P.; Waluk J.; Glasbeek M. (Sub)Picosecond Fluorescence Upconversion Studies of Intermolecular Proton Transfer of Dipyrido[2,3-*a*:3′,2′-*i*]Carbazole and Related Compounds. J. Phys. Chem. A 2000, 104, 7167–7175. 10.1021/jp994253u.

[ref37] Chang K.-H.; Peng Y.-C.; Su K.-H.; Lin Y.-H.; Liu J.-C.; Liu Y.-H.; Hsu C.-H.; Yang H.-C.; Chou P.-T. Long-Range Hydrogen-Bond Relay Catalyses the Excited-State Proton Transfer Reaction. Chem. Sci. 2023, 14, 7237–7247. 10.1039/D3SC01441J. 37416704 PMC10321479

[ref38] Nandi A.; Laude G.; Khire S. S.; Gurav N. D.; Qu C.; Conte R.; Yu Q.; Li S.; Houston P. L.; Gadre S. R.; Richardson J. O.; Evangelista F. A.; Bowman J. M. Ring-Polymer Instanton Tunneling Splittings of Tropolone and Isotopomers Using a Δ-Machine Learned CCSD(T) Potential: Theory and Experiment Shake Hands. J. Am. Chem. Soc. 2023, 145, 9655–9664. 10.1021/jacs.3c00769. 37078852 PMC10161208

[ref39] Vealey Z. N.; Foguel L.; Vaccaro P. H. Spectral Signatures of Proton-Transfer Dynamics at the Cusp of Low-Barrier Hydrogen Bonding. J. Phys. Chem. Lett. 2018, 9, 4949–4954. 10.1021/acs.jpclett.8b02199. 30101590

[ref40] Videla P. E.; Foguel L.; Vaccaro P. H.; Batista V. S. Proton-Tunneling Dynamics Along Low-Barrier Hydrogen Bonds: A Full-Dimensional Instanton Study of 6-Hydroxy-2-Formylfulvene. J. Phys. Chem. Lett. 2023, 14, 6368–6375. 10.1021/acs.jpclett.3c01337. 37418693

[ref41] van Thor J. J.; Champion P. M. Photoacid Dynamics in the Green Fluorescent Protein. Annu. Rev. Phys. Chem. 2023, 74, 123–144. 10.1146/annurev-physchem-091422-102619. 36696586

[ref42] Litman Y.; Richardson J. O.; Kumagai T.; Rossi M. Elucidating the Nuclear Quantum Dynamics of Intramolecular Double Hydrogen Transfer in Porphycene. J. Am. Chem. Soc. 2019, 141, 2526–2534. 10.1021/jacs.8b12471. 30648386 PMC6728096

[ref43] Smedarchina Z.; Siebrand W.; Fernandez-Ramos A. Entanglement and Co-Tunneling of Two Equivalent Protons in Hydrogen Bond Pairs. J. Chem. Phys. 2018, 148, 102307 10.1063/1.5000681. 29544290

